# A CONTENT ANALYSIS OF NATIONAL AFRICAN AMERICAN REPARATION COMMISSION’S REPARATIONS PLAN FOR OLDER BLACK AMERICANS

**DOI:** 10.1093/geroni/igad104.3138

**Published:** 2023-12-21

**Authors:** Jocelyn Brown

**Affiliations:** University of Maryland Baltimore, Baltimore, Maryland, United States

## Abstract

This policy content analysis critically examined the National African American Reparations Commission’s (NAARC) 10-Point Plan for Reparations. As the first comprehensive plan for reparations, the latest revision has gained attention for its potential to rectify socioeconomic inequality. However, the degree to which the plan addresses the nuanced challenges faced by older Black Americans (e.g., higher rates of poverty) is unclear. Therefore, the objectives of this analysis were to: a.) assess the plan’s potential to reduce inequality among older Black Americans, and b.) evaluate its capacity to address inequality over the life course and subsequent generations. Before the qualitative analysis began, a coding scheme was developed surrounding cumulative disadvantage theory. For example, codes addressing economic disparities, healthcare barriers, and intergenerational wealth transfer were developed. From there, the 12-page document was deductively coded by the author using this scheme. The findings suggest that the NAARC’s 2023 revision is considerably comprehensive, providing strategies to address socioeconomic inequality on structural levels (e.g., funds for entrepreneurial development, criminal justice reform). Even so, the plan needs work to fully address the needs of today’s older Black adults. To maximize the plan’s impact on older Black Americans, the NAARC could incorporate long-term care provisions, family-centered support systems, and long-term services and supports. This analysis has significant implications for the NAARC’s plan and continued fight for reparations. To maximize its impact on older Black Americans and promote intergenerational equity, policymakers should consider inclusive provisions for a demographic most directly haunted by the legacies of slavery and Jim Crow.

